# The Effect of Remote Ischemic Preconditioning on Serum Creatinine in Patients Undergoing Partial Nephrectomy: A Randomized Controlled Trial

**DOI:** 10.3390/jcm10081636

**Published:** 2021-04-12

**Authors:** Jaeyeon Chung, Min Hur, Hyeyeon Cho, Jinyoung Bae, Hyun-Kyu Yoon, Ho-Jin Lee, Young Hyun Jeong, Youn Joung Cho, Ja Hyeon Ku, Won Ho Kim

**Affiliations:** 1 Department of Anesthesiology and Pain Medicine, Seoul National University Hospital, College of Medicine, Seoul National University, 101 Daehak-Ro, Jongno-Gu, Seoul 03080, Korea; jychung1991@gmail.com (J.C.); bdbd7799@gmail.com (H.C.); baejy88@gmail.com (J.B.); hyunkyu18@gmail.com (H.-K.Y.); zenerdiode03@gmail.com (H.-J.L.); yhhh1130@naver.com (Y.H.J.); mingming7@gmail.com (Y.J.C.); 2 Department of Anesthesiology and Pain Medicine, School of Medicine, Ajou University, Suwon 16499, Korea; nuage1220@gmail.com; 3 Department of Urology, National University Hospital, College of Medicine, Seoul National University, 101 Daehak-Ro, Jongno-Gu, Seoul 03080, Korea; kukuro70@snu.ac.kr

**Keywords:** remote ischemic preconditioning, partial nephrectomy, acute kidney injury, urinary biomarker

## Abstract

Renal function declines after partial nephrectomy due to ischemic reperfusion injury induced by surgical insult or renal artery clamping. The effect of remote ischemic preconditioning (RIPC) on reducing renal injury after partial nephrectomy has not been studied regarding urinary biomarkers. Eighty-one patients undergoing partial nephrectomy were randomly assigned to either RIPC or the control group. RIPC protocol consisted of four cycles of five-min inflation and deflation of a blood pressure cuff to 250 mmHg. Serum creatinine levels were compared at the following time points: preoperative baseline, immediate postoperative, on the first and third days after surgery, and two weeks after surgery. The incidence of acute kidney injury, other surgical complication rates, and urinary biomarkers, including urine creatinine, β-2 microglobulin, microalbumin, and N-acetyl-beta-D-glucosaminidase were compared. Split renal functions measured by renal scan were compared up to 18 months after surgery. There was no significant difference in the serum creatinine level on the first postoperative day (median (interquartile range) 0.87 mg/dL (0.72–1.03) in the RIPC group vs. 0.92 mg/dL (0.71–1.12) in the control group, *p* = 0.728), nor at any other time point. There was no significant difference in the incidence of acute kidney injury. Secondary outcomes, including urinary biomarkers, were not significantly different between the groups. RIPC showed no significant effect on the postoperative serum creatinine level of the first postoperative day. We could not reveal any significant difference in the urinary biomarkers and clinical outcomes. However, further larger randomized trials are required, because our study was not sufficiently powered for the secondary outcomes.

## 1. Introduction

Remote ischemic preconditioning (RIPC) refers to applying one or more cycles of brief, nonlethal ischemia and reperfusion to a distant organ or tissue that is reported to protect distant major organs, including the heart, against acute ischemic insult [1]. Many clinical trials were reported under various clinical settings, proving the renal protective effect of RIPC [2,3]. Recently reported meta-analyses found that RIPC could mitigate the risk of acute kidney injury (AKI) in cardiac or vascular interventions [4,5]. However, there is still controversy regarding the effect of RIPC on postoperative renal dysfunction, depending on the type of surgery. A previous multicenter randomized trial reported RIPC could protect from the risk of postoperative AKI and hemodialysis after cardiac surgery [2]. Meanwhile, a recent Cochrane review reported that RIPC did not result in significant differences in terms of the serum creatinine, the incidence of AKI, and the requirement for dialysis in patients undergoing any interventions that could result in ischemic renal injury [6].

Partial nephrectomy is now regarded as a surgical modality of choice for localized small renal cell cancer [7]. By preserving the normal renal parenchyma, partial nephrectomy significantly reduced the risk of postoperative AKI, new-onset chronic kidney disease, and renal functional decline, compared to radical nephrectomy [8]. However, even after partial nephrectomy, the renal function significantly declines. The incidence of AKI after partial nephrectomy was reported as up to 39~51% [9]. Most of the reported risk factors for this functional decline are nonmodifiable. Although functional recovery after partial nephrectomy is mainly determined by parenchymal volume preservation [10], ischemic renal injury seems to be the main pathophysiology of AKI. During partial nephrectomy, the renal vascular pedicle is usually temporarily clamped, resulting in ischemia-reperfusion injury (IRI).

We assumed that RIPC could mitigate IRI and, therefore, may reduce renal ischemic injury in patients undergoing partial nephrectomy. Two previous randomized controlled trials evaluated the effect of RIPC during partial nephrectomy [11,12]. These trials reported the protective effect of RIPC on postoperative renal function, but one study did not measure biomarkers [11], and another other study measured the serum biomarkers of cystatin C and neutrophil gelatinase-associated lipocalin (NGAL) during the immediate postoperative period only [12]. Other urinary biomarkers, including urine creatinine, microalbumin, β-2 microglobulin, and N-acetyl-beta-D-glucosaminidase (NAG), were not studied in previous studies [13,14]. The glomerular filtration rate (GFR) and split function of a single kidney can also be measured by technetium diethylene triamine pentaacetic acid (99mTc-DTPA) radionuclide scintigraphy [15]. As AKI is associated with chronic kidney disease [16], the long-term effect of RIPC on GFR measured by radionuclide scintigraphy needs to be studied.

Therefore, in this randomized controlled trial, we tested the hypothesis that RIPC could reduce renal IRI during partial nephrectomy, thereby reducing the elevation in serum creatinine, as well as urinary biomarkers of acute kidney injury. We aimed to evaluate the effect of RIPC on postoperative renal function in terms of serum creatinine, GFR measured by scintigraphy, and urinary biomarkers.

## 2. Materials and Methods

### 2.1. Trial Design and Participants

The Institutional Review Board of Seoul National University Hospital (101, Daehak-ro, Jongro-gu, Seoul, Republic of Korea, Chairperson Yun-Chul Hong, approval number: 1707-087-870, protocol version 2.1) approved this prospective single-center, double-blinded randomized trial on 27 August 2018. Our study was registered on http://www.clinicaltrials.gov (NCT03273751, first posted on 6 September 2017), and our study protocol was previously published [17]. This study was performed in accordance with the Good Clinical Practice Guidelines and stuck to the applicable Consolidated Standards of Reporting Trials (CONSORT) guidelines. We conducted this study at a single tertiary-center university hospital. Our coauthors obtained written informed consent from all participants.

Eligible participants included adults (≥20 years of age) undergoing elective laparoscopic or robot-assisted or open partial nephrectomy. Patients with normal contralateral renal function were included. Renal function was confirmed with the preoperative split renal function of >40% by 99mTc-DTPA radionuclide scintigraphy and was compared with the function of the kidney on the surgery.

We excluded the patients with any of the following comorbidities: clinically relevant peripheral vascular disease that affects the upper arms where we applied the RIPC; severe cardiopulmonary diseases (ejection fraction of the left ventricle <40%, heart failure, valvular or ischemic heart disease, forced expiratory volume in 1 s of <40% of the predicted value, or chronic obstructive pulmonary disease); hepatic failure (bilirubin level of >1.2 mg/dL or prothrombin time international normalized ratio of >2.0); and baseline chronic kidney disease (estimated glomerular filtration rate of <30 mL/min/1.73 m^2^ of the body–surface area or preoperative serum creatinine level > 1.4 mg/dL).

### 2.2. Randomization and Blinding

We randomly assigned the initially enrolled participants to either the RIPC or control group in a 1:1 ratio according to computer-generated random numbers in block sizes of 4 or 6 (http://www.sealedenvelope.com (accessed on 20 January 2021)). We concealed the group allocations from the investigators using opaque envelopes. A third party independent of the study conducted the random assignment, and the assignment sequences were not disclosed until the study ended. On the operation day, the opaque envelope containing the group assignment was delivered to an anesthesiologist who was responsible for patient care and the implementation of the RIPC but was not involved in the study. The participants, post-anesthesia care staff, urologic surgeons, data collectors, and outcome assessors were also blinded to the group assignment to minimize potential sources of bias.

### 2.3. Study Protocol

Anesthesia was induced with 1.5–2 mg/kg of intravenous propofol and a continuous infusion of remifentanil (effect-site concentration of 2–5 ng/mL) using a target-controlled infusion pump (Orchestra^®^; Fresenius Kabi, Bad Homburg, Germany). Intravenous rocuronium 0.6 mg/kg was administered to facilitate endotracheal intubation. Desflurane or sevoflurane was used to maintain general anesthesia using a 1–1.5 minimum alveolar concentration.

After the patients take a left or right upper lateral position, an NIRS sensor (INVOS™ 5100C Cerebral/Somatic Oximetry Adult Sensor, Medtronic, Minneapolis, MN, USA) was applied under ultrasound guidance directly to the flank area that overlays the opposite kidney not undergoing surgery to monitor the renal regional oxygen saturation (rSO_2_) [18]. The renal rSO_2_ was continuously monitored with an NIRS Oximeter until the end of the surgery. The values of the rSO_2_ were recorded at intervals of 10 min during the surgery.

After the anesthesia induction and before cross-clamping the renal artery, participants assigned to the RIPC group received the RIPC protocol on the dependent upper arm in the lateral position. The protocol of RIPC was conducted using an automated cuff inflator, which comprised four cycles of 5-min inflation of a blood pressure cuff to 250 mmHg or, at least, to a pressure 50 mmHg higher than the participants’ systolic arterial pressure, followed by 5-min deflation of the cuff. In participants assigned to the control group, a blood pressure cuff was also placed on the upper arm but without inflating the cuff during the study period. An anesthesiologist who was independent of this study conducted the RIPC protocol.

### 2.4. Study Outcomes

Our primary outcome was the serum creatinine level on the first postoperative day (POD) as a surrogate for the postoperative renal function. Our secondary outcomes included the postoperative serum creatinine at other time points, such as the baseline, immediate postoperative period, third POD, and two weeks after surgery. Other secondary outcomes included the incidence of postoperative AKI and urinary biomarkers such as urine creatinine, microalbumin, β-2 microglobulin, and NAG (N-acetyl-beta-D-glucosaminidase) measured immediately and on one day and two weeks after partial nephrectomy [13,14]. We adjusted the urinary NAG as a ratio to the urinary creatinine concentration, because the adjusted urinary NAG showed less variability than the urinary NAG excretion itself related to volume or time [19]. Postoperative AKI was diagnosed based on the serum creatinine criteria of Kidney Disease Improving Global Outcomes (KDIGO) criteria [20]. We defined postoperative AKI according to the postoperative increase in the serum creatinine level (stage 1: >1.5-fold or >0.3 mg/dL increase, stage 2: >2-fold, and stage 3: > 3-fold increase or >4.0-mg/dL increase or initiation of renal replacement therapy) within 2 weeks after surgery. The most recent preoperative serum creatinine measured within 1 month before surgery was used as a baseline. Estimated GFR (eGFR) was measured immediately and at one, three days, and two weeks after partial nephrectomy. Renal rSO_2_ of the non-operated kidney measured at 10-min intervals from anesthesia induction to the end of surgery were also investigated as secondary outcomes. eGFR was calculated with the abbreviated isotope dilution mass spectrometry Modification in Diet and Renal Disease Study equation: eGFR (mL/min/1.73 m^2^) = 175 × (serum creatinine) − 1.154 × (age) − 0.203 × 0.742 (if female) × 1.212 (if black) [21]. Additionally, the GFR obtained by 99mTc-DTPA renal scintigraphy was measured at the preoperative baseline and at 6 and 12~18 months postoperatively.

We collected the baseline characteristics, including age, sex, body mass index, preoperative baseline eGFR, serum creatinine concentration, hemoglobin, 99mTc-DTPA renal scintigraphy, comorbidities, medication history, smoking, and alcohol consumption. We also collected surgery and anesthesia-related parameters, including the radius, exophytic/endophytic properties, nearness of tumor to collecting system or sinus, anterior/posterior, hilar, location relative to polar lines (R.E.N.A.L.) nephrometry score [22]. Postoperative data were assessed, including the length of hospital and intensive care unit (ICU) stay, incidences of postoperative complications, including postoperative wound infection, reoperation, myocardial infarction, venous thromboembolism, and cerebrovascular accidents.

### 2.5. Statistical Analysis

Data were presented as the mean (standard deviation), median (interquartile range) or the incidence (%). The normality of the data distribution was tested by the Kolmogorov–Smirnov test. To compare the baseline characteristics and the outcome variables between the two groups, the Mann–Whitney *U* test or Student’s *t*-test was used for continuous parameters, depending on the distribution of data, and Fisher’s exact test or chi-square test was used for the incidence variables, depending on the expected counts. To compare the time-dependent values of eGFR, serum creatinine, and urinary biomarkers, as well as renal rSO_2_ of the opposite kidney, repeated-measures analysis of variance or linear mixed model was used, depending on the presence of missing data to compare between the groups. Multiple imputations using the Markov chain Monte Carlo algorithm were used to handle the missing values. As a post-hoc analysis, a multivariable logistic regression analysis was performed to evaluate whether the RIPC has an independent association with the risk of AKI. Potential confounding factors known to affect the risk of AKI, such as patient characteristics (age, sex, history of hypertension, diabetes mellitus, renal insufficiency, and congestive heart failure), and the surgical variables shown in Table 1 were used as the covariates. A backward stepwise variable selection process was used with a cutoff of *p* < 0.10. We also performed a subgroup analysis based on the surgical techniques and R.E.N.A.L. nephrometry score. We compared the incidence of AKI and risk differences in different types of partial nephrectomy and R.E.N.A.L. score subgroups.

Our sample size was determined as follows. We assumed that the serum creatinine levels on the first POD in the RIPC group were significantly lower than those in the control group by more than 0.35 mg/dL. At least 39 patients per group were required, with 80% power and a two-sided alpha error of 0.05. For this calculation, the mean value of our primary outcome of 1.60 mg/dL and standard deviation of 0.54 mg/dL, according to our pilot data, were used. Considering the 10% drop-out rate, we determined the final sample size as 43 patients per group. G*power (version 3.1.9.2, Universität Düsseldorf, Düsseldorf, Germany) was used to calculate the sample size.

*p* < 0.05 was considered statistically significant. To reduce type I errors, Bonferroni correction for multiple measurements was used. Data were analyzed using SPSS software (SPSS version 22.0, Chicago, IL, USA).

## 3. Results

**Figure 1:**
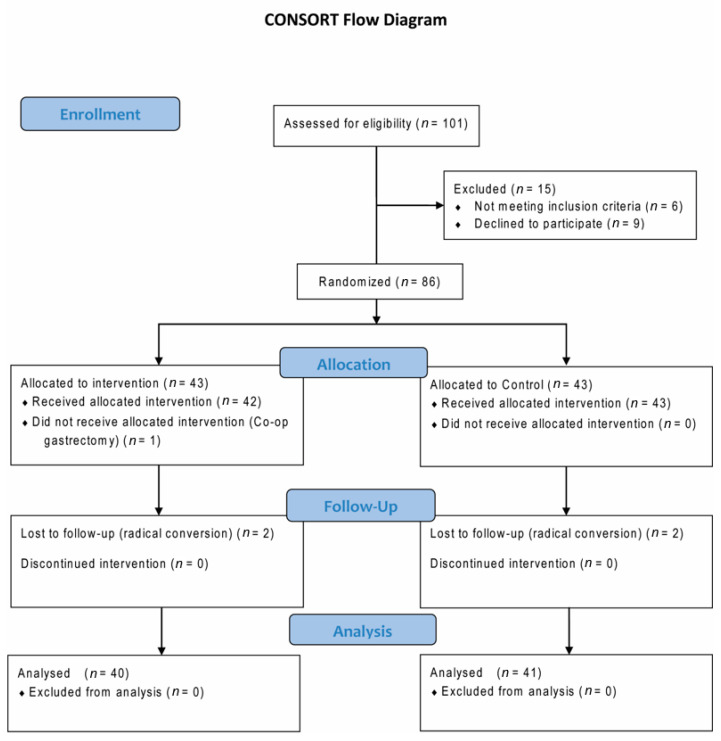
Consolidated Standards of Reporting Trials (CONSORT) flowchart.

Among the 101 patients assessed for eligibility, 86 patients were initially enrolled in this study. The patients whose operation plan was changed to radical nephrectomy (*n* = 4) and other concomitant surgical procedures (*n* = 1) were excluded from the analysis (Figure 1). There was no relevant complication associated with the anesthesia or RIPC protocol.

Table 1 shows the demographic data and perioperative parameters. Table 2 shows the effect size of our primary outcome. There was no significant difference in the serum creatinine level between the two groups on the first postoperative day (median (interquartile range) 0.87 mg/dL (0.72–1.03) in the RIPC group vs. 0.92 mg/dL (0.71–1.12) in the control group, *p* = 0.728).

**Figure 2:**
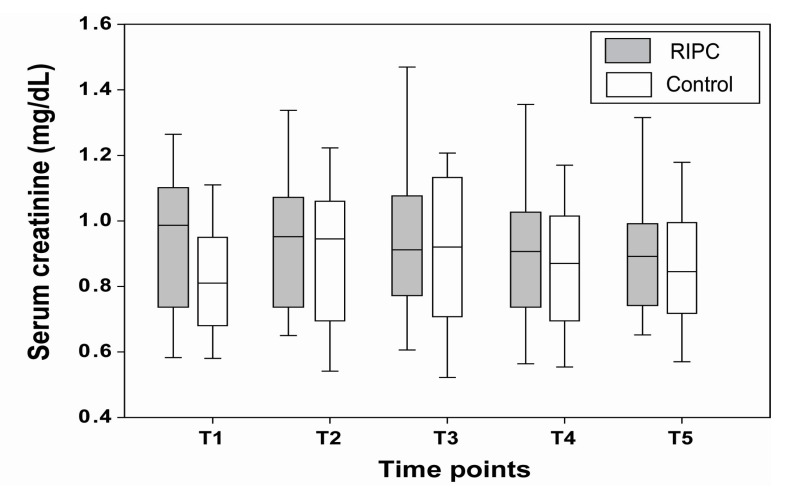
Comparison of time-dependent changes in the serum creatinine between the RIPC and control groups. Time points were defined as preoperative (T1), at post-anesthesia care unit (T2), postoperative day 1 (T3), postoperative day 3 (T4), and two weeks after surgery (T5).

**Figure 3:**
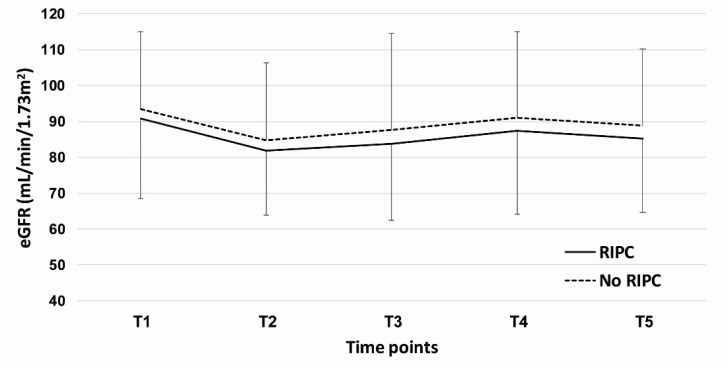
Comparison of the time-dependent changes in the estimated glomerular filtration rate between the RIPC and control groups. The time points were defined as preoperative (T1), at post-anesthesia care unit (T2), postoperative day 1 (T3), postoperative day 3 (T4), and two weeks after surgery (T5).

Figure 2 shows time-dependent changes in the serum creatinine, and there was no significant difference between the RIPC and control groups at all five time points. Figure 3 shows the time-dependent changes in the eGFR, and there were no significant differences between the two groups at all the time points.

Table 3 shows several secondary clinical outcomes between the RIPC and control groups. The incidence of AKI was not significantly different between the two groups (RIPC 12.2% (5/41) vs. Control 17.5% (7/40), *p* = 0.331), and all of them were stage I AKI. The length of hospital stay and postoperative transfusion rate were similar in both groups. The postoperative complication rate, described as a Clavien–Dindo classification, was not significantly different between the two groups.

The split renal function and GFR measured by a 99mTc-DTPA scan at baseline and 6 and 12~18 months after surgery are summarized in Supplementary Figures S1–S6. However, there was no significant difference between the two groups at each time point.

Supplementary Figure S7 shows time-dependent changes in the rSO_2_ of the contralateral kidney during surgery. There was no significant difference in the baseline and intraoperative rSO_2_ between the two groups. Supplementary Figure S8 shows time-dependent changes in the serum high-sensitivity C-reactive protein (hs-CRP), and there were no significant differences in the four time points (baseline, POD 1, POD 3, and two weeks after surgery). Supplementary Figures S9–S12 show time-dependent changes in the urinary biomarkers, including the urinary creatinine, microalbumin, β-2 microglobulin, and NAG measured at the baseline, immediately postoperative, and one day and two weeks after surgery. There were no significant differences between the two groups’ urinary biomarkers at each time point.

The multivariable logistic regression analysis showed that radiocontrast used within one month (odds ratio: OR (95% confidence interval) = 5.73 (1.05–31.3), *p* = 0.044), R.E.N.A.L. score (2.37 (1.25–4.52), *p* = 0.009), and preoperative hemoglobin (2.11 (1.17–3.80), *p* = 0.013) are statistically significant parameters associated with postoperative AKI (Table 4).

A subgroup analysis of the incidence of AKI according to the type of partial nephrectomy and R.E.N.A.L. score showed no significant differences between the RIPC and control groups (Table 5).

## 4. Discussion

Patients who undergo partial nephrectomy are likely to develop postoperative renal functional decline due to both renal parenchymal reduction and ischemia–reperfusion injury of the remaining parenchyma. In this prospective randomized trial, we evaluated the effect of RIPC on the postoperative renal function in patients undergoing partial nephrectomy. The postoperative renal function was evaluated by various outcomes, including the serum creatinine value, the incidence of acute kidney injury, surgical complication rate, urinary biomarkers, and split renal function measured by a renal radionuclide scan. Although our study was powered only on the primary outcome of serum creatinine of the first postoperative day, all our primary and secondary outcomes were not significantly different between the groups. RIPC seems to have no effect on the postoperative renal function in this patient group with high-risk postoperative renal dysfunction.

The renal protective effect of RIPC has been extensively studied in the various types of procedures, including cardiac surgery or procedure [23], major vascular surgery [24], and percutaneous coronary intervention (PCI) [25], while the effect for renal protection is inconclusive. Partial nephrectomy is a standard treatment for localized small kidney tumors [7]. Although the incidence is much lower than radical nephrectomy, AKI still occurs in up to 39-51% of patients after partial nephrectomy [8,9]. AKI developed after partial nephrectomy is not only a short-term problem, because AKI can be directly linked to the progression of chronic kidney disease [26].

The optimal protocol and regimen of RIPC, including the timing, number of ischemia/reperfusion cycles, and duration of each ischemic period, have not yet been established. Important variables in the regimen of RIPC include the optimal duration of the ischemia, the number of cycles repeated, and the site of application of the ischemia [27]. According to the Cochrane review of ischemic preconditioning (IPC) for the reduction of renal ischemia–reperfusion injury [6], the most common sites to which the IPC is applied are the upper and lower extremities, and three or four cycles of ischemia and reperfusion are used at five-min intervals. As the muscle mass is different between the upper and lower limbs, the choice of the limb may influence the effect of RIPC. To provide enough ischemic insult to the limb to maximize the effect of RIPC, we decided to apply four cycles of five-min ischemia and five-min reperfusion [27].

Regarding the inflation pressure of the cuff, a threshold of 200 mmHg has commonly been used in previous studies to induce ischemia of the upper extremities. However, a 200-mmHg inflation of the blood pressure cuff might be insufficient to occlude the arterial blood flow of the upper limb in patients with chronic hypertension [1]. In addition, there were no complications induced by the inflation of the cuffs in studies applying RIPC in the upper limb with inflation pressures of 300 mmHg, and studies using high inflation pressure reported significant protective effects of RIPC [28].

The postoperative serum creatinine level and other secondary outcomes were not significantly different between RIPC and the control groups. These insignificant results can be explained as follows. First, the overall AKI incidence of our study was 14.8%, which was lower than previous studies. The AKI incidence was reported to be significantly different between the surgical modalities, favoring robot surgery compared to open and even laparoscopic surgery [20,29]. Compared to the previous studies that included laparoscopic surgery only, robotic partial nephrectomy comprised 48% in our study population, which could mask the renal-protective effect of RIPC in this study. Second, propofol was used as an anesthesia induction agent in our study. Previous studies showed that propofol could suppress the protective effect of RIPC on renal dysfunction [30]. In a previous meta-analysis of RIPC in adult cardiac surgery, the renal-protective effect of RIPC was seen in a specific subgroup that used volatile anesthetics without propofol [23]. Propofol was used for the anesthesia induction in our study, and this could also mitigate the protective effect of RIPC.

To our knowledge, two studies have evaluated the effect of RIPC in patients undergoing partial nephrectomy [11,12]. A previous randomized trial evaluated the effect of RIPC in patients undergoing laparoscopic partial nephrectomy [11]. RIPC consisted of three five-min cycles of right lower limb ischemia, while we used four five-min cycles of upper arm ischemia. The study included only laparoscopic surgery, while we enrolled patients undergoing both laparoscopic and open surgery. They found a significant difference in the percentage changes of differential GFR of the affected kidney at one month after surgery between the RIPC and the control groups. The decrease in differential GFR at one month after surgery was significantly lesser, and the retinol-binding protein levels measured at 24 h after surgery were significantly lower in the RIPC group compared to the control group. However, they could not find a consistent significant difference at six months after surgery, which means the lack of a long-term effect. We could not find any difference in the split renal function and differential GFR up to 18 months after surgery. We also could not find any difference in our urinary biomarkers up to two weeks after surgery. These differences may be due to different patient demographics, comorbidities, general anesthetic agents, and types of surgery, all of which could influence the effect of RIPC [27]. Both our and the previous study could not find any significant differences in the total renal function measured by the serum creatinine or eGFR up to six months after surgery. Both studies failed to reveal any long-term effects of RIPC in terms of the split or total renal function and biomarker of renal injury.

Recently published, the other study evaluated the effects of late and early RIPC, which were conducted 24 h after surgery and after the induction of anesthesia, respectively [12]. Serum NGAL and cystatin C, as well as GFR zero, two, and six hours after surgery, were measured in 65 patients undergoing laparoscopic partial nephrectomy. They reported that the serum NGAL (neutrophil gelatinase-associated lipocalin) and cystatin C were significantly lower in both RIPC groups.

Our multivariable logistic regression analysis showed that the hemoglobin, R.E.N.A.L. nephrometry score, and radiocontrast use were significant risk factors of AKI development. The association between a low hemoglobin level and AKI has been reported in previous studies [31]. The R.E.N.A.L. nephrometry score is a standard anatomic classification system of kidney tumors that is associated with postoperative surgical complications [22]. The R.E.N.A.L score is useful in predicting postoperative renal function after partial nephrectomy [32]. Radiocontrast-induced nephropathy is one of the leading causes of AKI [33]. RIPC has been widely studied to reduce radiocontrast-induced AKI, with conflicting results for its renal protective effect [34]. Interestingly, the effect of RIPC on contrast-induced AKI seems different from AKI caused by ischemia–reperfusion injury, according to a recent meta-analysis [35]. In our study population, as the radiocontrast was used for computed tomography (CT) and ischemia–reperfusion injury developed due to surgical insult, the effect of RIPC was not significant both for radiocontrast-induced AKI and AKI associated with ischemia–reperfusion injury.

We monitored the rSO_2_ near the opposite kidney not undergoing surgery based on previous study results that renal rSO_2_ desaturation was associated with AKI in cardiac surgery [18]. There was no significant difference in the rSO_2_ between the RIPC and the control groups in our study. Previous studies of pediatric populations have also reported the potential benefits of renal rSO_2_ monitoring for predicting postoperative AKI [36,37]. As the exact penetration depth of the NIRS sensor is unknown, our sensor may inaccurately detect the low renal oxygen saturation.

Our study should be interpreted under several limitations. First, our primary outcome was postoperative serum creatinine. To evaluate any difference in AKI incidence, much more participants will be required. If we calculate the sample size to detect a 10% difference in the incidence of AKI, assuming the incidence of AKI to be 20%, more than 200 patients would be required in each group. Furthermore, the difference in serum creatinine on the first postoperative day of 0.35 mg/dL was greater than a 0.3-mg/dL increase for defining stage 1 AKI, according to the KDIGO criteria [20]. Therefore, our study results should be interpreted under a possible power shortage. Second, as described in the discussion, propofol was used as an induction agent in this study, which could suppress the protective effect of RIPC on renal dysfunction [30,38]. Third, urinary creatinine, microalbumin, ß-2 microglobulin, and NAG were chosen as biomarkers of renal injury in the present study. There are other biomarkers with better performances, such as serum cystatin C and NGAL or the urine tissue inhibitor of metalloproteinase 2 (TIMP-2) and insulin-like growth factor-binding protein 7 (IGFBP7) [12,39]. Measuring these biomarkers would enhance the sensitivity to detect and compare renal injuries.

## 5. Conclusions

Our randomized controlled trial showed that RIPC has no significant effect on the postoperative serum creatinine level of the first postoperative day. We could not find any significant effects of the other secondary outcomes, including the incidence of acute kidney injury, split renal function, and biomarkers of renal injury up to six months after surgery. However, as our study was powered up to the serum creatinine level of postoperative day one, further studies with sufficient power are still required to confirm the null effect of RIPC on the renal function after partial nephrectomy.

## Figures and Tables

**Table 1:** Patient characteristics and perioperative parameters.

Variables	RIPC Group (*n* = 41)	Control Group (*n* = 40)	Standardized Difference (95% Confidence Interval)
Demographic data			
Age, y	63 (52–72)	64 (56–69)	0.47 (−0.12 to 0.91)
Female, *n* (%)	13 (31.7)	13 (32.5)	0.02 (−0.42 to 0.45)
Body mass index, kg/m^2^	25.0 (23.3–26.4)	24.0 (21.1–26.6)	0.31 (−0.13 to 0.75)
Baseline medical status			
Hypertension, *n*	15 (36.6)	15 (37.5)	0.02 (−0.42 to 0.45)
Diabetes mellitus, *n*	9 (22.0)	6 (15.0)	0.18 (−0.26 to 0.62)
Hypercholesterolemia, *n*	9 (22.0)	2 (5.0)	0.51 (0.07 to 0.96)
Coronary artery disease, *n*	3 (7.3)	0 (0)	0.40 (−0.04 to 0.84)
Cerebrovascular accident, *n*	1 (2.4)	0 (0)	0.22 (−0.21 to 0.66)
Arrhythmia, *n*	1 (2.4)	1 (2.5)	0.00 (−0.43 to 0.44)
Chronic obstructive pulmonary disease, *n*	-	-	-
Asthma, *n*	1 (2.4)	1 (2.5)	0.00 (−0.43 to 0.44)
ASA physical status classification (1/2/3), *n*	16 (39.0)/23 (56.1)/ 2 (4.9)	21 (52.5)/20 (50.0)/0 (0)	0.37 (−0.07 to 0.81)
Radiocontrast administration within one month, *n*	11 (26.8)	13 (32.5)	0.12 (−0.31 to 0.56)
Number of antihypertensive agents, *n*	0 (0–1)	0 (0–1)	0.38 (−0.10 to 0.85)
Angiotensin-converting enzymes, *n*	1 (2.6)	0 (0)	0.23 (−0.21 to 0.67)
Smoking, pack year	0 (0–0)	0 (0–0)	0.32 (−0.12 to 0.76)
Baseline laboratory findings			
Hemoglobin, g/dL	14.1 (13.0–15.1)	14.2 (13.0–15.2)	0.11 (−0.33 to 0.55)
Serum albumin, g/dL	4.4 (4.2–4.5)	4.6 (4.3–4.7)	0.40 (−0.04 to 0.84)
Total cholesterol, mg/dL	168 (153–217)	188 (167–219)	0.34 (−0.097 to 0.78)
Blood glucose, mg/dL	102 (96–122)	105 (96–122)	0.03 (−0.40 to 0.47)
Hemoglobin A1c	5.7 (5.4–6.5)	5.5 (5.3–5.9)	0.31 (−0.17 to 0.80)
Erythrocyte sedimentation rate	12.0 (6.8–23.5)	10.5 (4.0–17.5)	0.42 (−0.04 to 0.88)
Surgical parameters			
Surgery type, *n*			
Laparoscopic/Robot-assisted/Open	8 (19.5)/21 (51.2)/ 12 (29.3)	2 (5.0)/18 (45.0)/ 20 (50.0)	0.56 (0.11 to 1.00)
Clinical stage, *n*			
T1a/ T1b	33 (80.5)/6 (14.6)	34 (85.0)/6 (15.0)	-
T2a/ T2b	1(2.4)/-	-/-	
T3a/ T3b / T3c	-/-/-	-/-/-	
N 0/1	41/-	40/-	-
M 0/1	41/-	40/-	-
R.E.N.A.L. score	5 (4–8)	7 (5–8)	0.35 (−0.09 to 0.79)
Low (4–6)	25 (61.0)	17 (42.5)	0.46 (0.02 to 0.91)
Intermediate (7–9)	16 (39.0)	24 (60.0)	
High (10–12)	-	-	
Tumor maximal diameter, cm	2.5 (2.0–3.4)	2.2 (1.6–3.6)	0.06 (−0.38 to 0.50)
Tumor location (anterior/posterior/neither)	14 (34.1)/20 (48.8)/ 7 (17.1)	17 (42.5)/14 (35.0)/7 (17.5)	0.28 (−0.16 to 0.72)
Operation time, min	100 (83–118)	110 (83–128)	0.04 (−0.39 to 0.48)
Renal ischemic time, min	17.0 (13.2–21.2)	17.0 (12.5–21.6)	0.01 (−0.44 to 0.45)
Anesthesia time, min	140 (115–160)	145(115–165)	0.14 (−0.30 to 0.57)
Preoperative DTPA renal scan			
Left split function, %	51 (47–53)	50 (47–52)	0.25 (−0.19 to 0.68)
Left GFR, mL/min/1.73 m^2^	44 (31–53)	43 (36–56)	0.15 (−0.29 to 0.59)
Right split function, %	49 (47–53)	50 (48–53)	0.30 (−0.14 to 0.74)
Right GFR, mL/min/1.73 m^2^	38 (33–50)	49 (39–55)	0.33 (−0.11 to 0.77)
Total GFR, mL/min/1.73 m^2^	82 (65–104)	93 (78–106)	0.25 (−0.19 to 0.69)
Normalized GFR, mL/min/1.73 m^2^	84 (66–102)	86 (72–110)	0.24 (−0.20 to 0.68)
Bleeding and transfusion amount			
pRBC transfusion, *n*	-	-	-
pRBC transfusion, unit	-	-	-
FFP transfusion, unit	-	-	-
Estimated blood loss, mL	100 (50–200)	150 (58–263)	0.26 (−0.18 to 0.70)
Anesthesia-related parameters			
Volatile anesthetics use, *n*			
Sevoflurane, *n*	9 (22.0)	9 (22.5)	0.01 (−0.42 to 0.45)
Desflurane, *n*	32 (78.0)	32 (80.0)	
Crystalloid administration, mL	800 (550–950)	875 (678–1063)	0.36 (−0.08 to 0.80)
Colloid administration, mL	-	-	-
Intraoperative urine output, mL	100 (50–200)	95 (50–155)	0.39 (−0.05 to 0.83)
Intraoperative arterial blood pressure			
Mean, mmHg	85 (81–91)	87 (78–94)	0.10 (−0.34 to 0.53
Maximum, mmHg	108 (102–118)	112 (102–120)	0.18 (−0.26 to 0.62)
Minimum, mmHg	67 (54–72)	68 (62–74)	0.40 (−0.05 to 0.83)
Intraoperative ephedrine dose, mg	5 (0–10)	0 (0–5)	0.19 (−0.25 to 0.62)
Intraoperative phenylephrine dose, mcg	0 (0–0)	0 (0–0)	0.17 (−0.27 to 0.61)
Vasopressor infusion during surgery	1 (2.4)	-	-

RIPC = remote ischemic preconditioning; ASA = American Society of Anesthesiologist; DTPA = diethylenetriamine pentaacetic acid; GFR = glomerular filtration rate; R.E.N.A.L. score = radius, exophytic/endophytic properties, nearness of tumor to collecting system or sinus, anterior/posterior, hilar, location relative to polar lines; and pRBC = packed red blood cells.

**Table 2:** Comparison of the secondary clinical outcomes between the RIPC and control groups.

Variables	RIPC Group (*n* = 41)	Control Group (*n* = 40)	*p*-Value	Difference in Medians (95% Confidence Interval)
Postoperative day one serum creatinine, mg/dL	0.87 (0.72–1.03)	0.92 (0.71–1.12)	0.728	0.0 (−0.11 to 0.13)

Data are presented as the median (Interquartile range). RIPC = remote ischemic preconditioning.

**Table 3:** Comparison of the secondary clinical outcomes between the RIPC and control groups.

Variables	RIPC Group (*n* = 41)	Control Group (*n* = 40)	*p*-Value	Difference in Medians or Risk (95% Confidence Interval)
Acute kidney injury, *n*	5 (12.2)	7 (17.5)	0.502	0.66 (0.19 to 2.27)
Length of hospital stay, days	5 (5–5)	5 (5–5)	0.348	0 (0 to 0)
Length of ICU stay, days	0 (0–0)	0 (0–0)	0.554	0 (0 to 0)
Postoperative pRBC transfusion, *n*	1 (2.4)	1 (2.4)	0.999	-
Postoperative complications, *n*	2 (4.9)	3 (7.3)	0.675	0.63 (0.10 to 4.00)
Bleeding, *n*	1 (2.4)	2 (5.0)	-	-
Wound dehiscence, *n*	-	1 (2.5)	-	-
Postoperative seizure, *n*	1 (2.4)	-	-	-
Clavien–Dindo classification, Grade 1/2/3/4/5	12(29.3)/1(2.4)/1(2.4) /-/-	8(20.0)/1(2.5)/2(5) /-/-	0.774	1.12 (0.44 to 2.83)

Data are presented as the median (Interquartile range) or number (%). Risk differences are for the RIPC group relative to the control group; differences are RIPC group–control group. POD = postoperative day, CI = confidence interval, RIPC = remote ischemic preconditioning, ICU = intensive care unit, and pRBC = packed red blood cells.

**Table 4:** Multivariable logistic regression analysis of the risk of acute kidney injury after partial nephrectomy.

Variables	Odds Ratio	95% Confidence Interval	*p*-Value
Radiocontrast use within 1 month	5.73	1.05–31.3	0.044
R.E.N.A.L. score	2.37	1.25–4.52	0.009
Preoperative hemoglobin (mg/dL)	2.11	1.17–3.80	0.013

The multivariable logistic regression analysis was performed using all the variables with *p* < 0.2 in the univariate logistic analysis. Backwards stepwise variable selection was used in the analysis with a cut-off *p*-value of less than 0.10. Nagelkerke R^2^ statistic was 0.478. Hosmer & Lemeshow goodness of fit test was not significant (*p* =0.675). R.E.N.A.L. score = nephrometry score (radius, exophytic/endophytic properties, nearness of tumor to collecting system or sinus, anterior/posterior, hilar, location relative to polar lines).

**Table 5:** Comparison of the incidence of acute kidney injury (AKI) between the RIPC and control groups according to the surgical modalities and R.E.N.A.L. score.

Variables	Incidence of AKI		*p*-Value	Risk Difference (95% Confidence Interval)
	RIPC (*n* = 41)	Control (*n* = 40)		
Type of Partial Nephrectomy (*n* = 81)				
Laparoscopic (*n* = 10)	2/8 (25.0)	-/2 (0.0)	0.999	-
Robot-assisted (*n* = 39)	1/21 (4.8)	3/18 (16.7)	0.318	0.25 (0.02 to 2.65)
Open (*n* = 32)	2/12 (16.7)	4/20 (20.0)	0.999	0.80 (0.12 to 5.20)
R.E.N.A.L. score				
Low (4–6) (*n* = 40)	1/25 (4.0)	-/15 (0.0)	0.999	-
Intermediate (7–9) (*n* = 40)	4/16 (25.0)	7/24 (29.2)	0.717	0.692 (0.15 to 3.3)
High (10–12) (*n* = 0)	-	-	-	

Data are presented as the number (%). RIPC = remote ischemic preconditioning and R.E.N.A.L. score = nephrometry score (radius, exophytic/endophytic properties, nearness of tumor to collecting system or sinus, anterior/posterior, hilar, location relative to polar lines).

## Data Availability

The data presented in this study are available on request from the corresponding author.

## Supplementary Materials

The following are available online at https://www.mdpi.com/article/10.3390/jcm10081636/s1: Supplementary Figure S1. Comparison of time-dependent changes of the left split renal function measured by 99mTc-DTPA renal scintigraphy between the RIPC and control groups. Supplementary Figure S2. Comparison of time-dependent changes of the left glomerular filtration rate measured by 99mTc-DTPA renal scintigraphy between the RIPC and control groups. Supplementary Figure S3. Comparison of time-dependent changes of the right split renal function measured by 99mTc-DTPA renal scintigraphy between the RIPC and control groups. Supplementary Figure S4. Comparison of time-dependent changes of the right glomerular filtration rate measured by 99mTc-DTPA renal scintigraphy between the RIPC and control groups. Supplementary Figure S5. Comparison of time-dependent changes of the total glomerular filtration rate measured by 99mTc-DTPA renal scintigraphy between the RIPC and control groups. Supplementary Figure S6. Comparison of time-dependent changes of the normalized glomerular filtration rate measured by 99mTc-DTPA renal scintigraphy between the RIPC and control groups. Supplementary Figure S7. Comparison of time-dependent changes of the regional O_2_ saturation (rSO_2_) during partial nephrectomy between the RIPC and control groups. Supplementary Figure S8. Comparison of time-dependent changes of high-sensitive C-reactive protein between the RIPC and control groups. Supplementary Figure S9. Comparison of time-dependent changes of urine creatinine between the RIPC and control groups. Supplementary Figure S10. Comparison of time-dependent changes of urine microalbumin between the RIPC and control groups. Supplementary Figure S11. Comparison of time-dependent changes of urine β-2 microglobulin between the RIPC and control groups. Supplementary Figure S12. Comparison of time-dependent changes of urine N-acetyl-beta-D-glucosaminidase (NAG) between the RIPC and control groups.
