# Supplementary material for: The Effect of Remote Ischemic Preconditioning on Serum Creatinine in Patients Undergoing Partial Nephrectomy: A Randomized Controlled Trial
Source: J Clin Med. 2021 Apr 12;10(8):1636. doi: 10.3390/jcm10081636 (PMC8069991; doi:10.3390/jcm10081636)
Supplement: Supplementary file 1 [file jcm-10-01636-s001.pdf]

Supplementary material

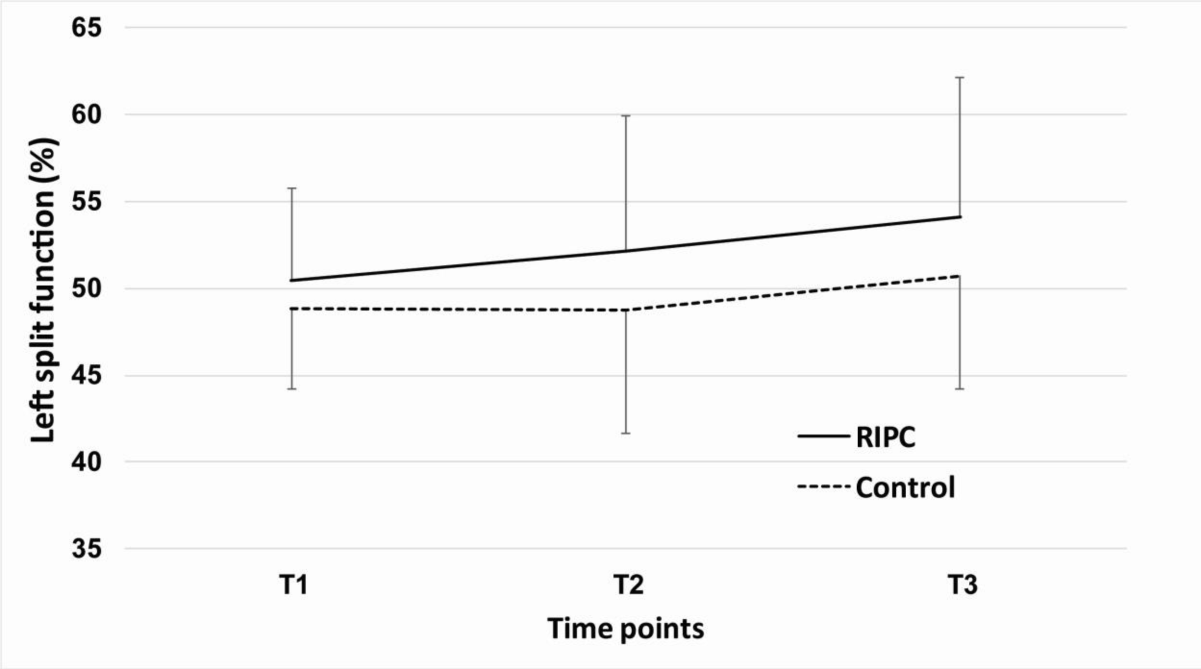

Supplementary Figure S1. Comparison of time dependent change of left split renal function measured by 99mTc-DTPA renal scintigraphy between RIPC and control group. The time points were defined as preoperative (T1), 6 month after surgery (T2) and 12~18 month after surgery (T3).

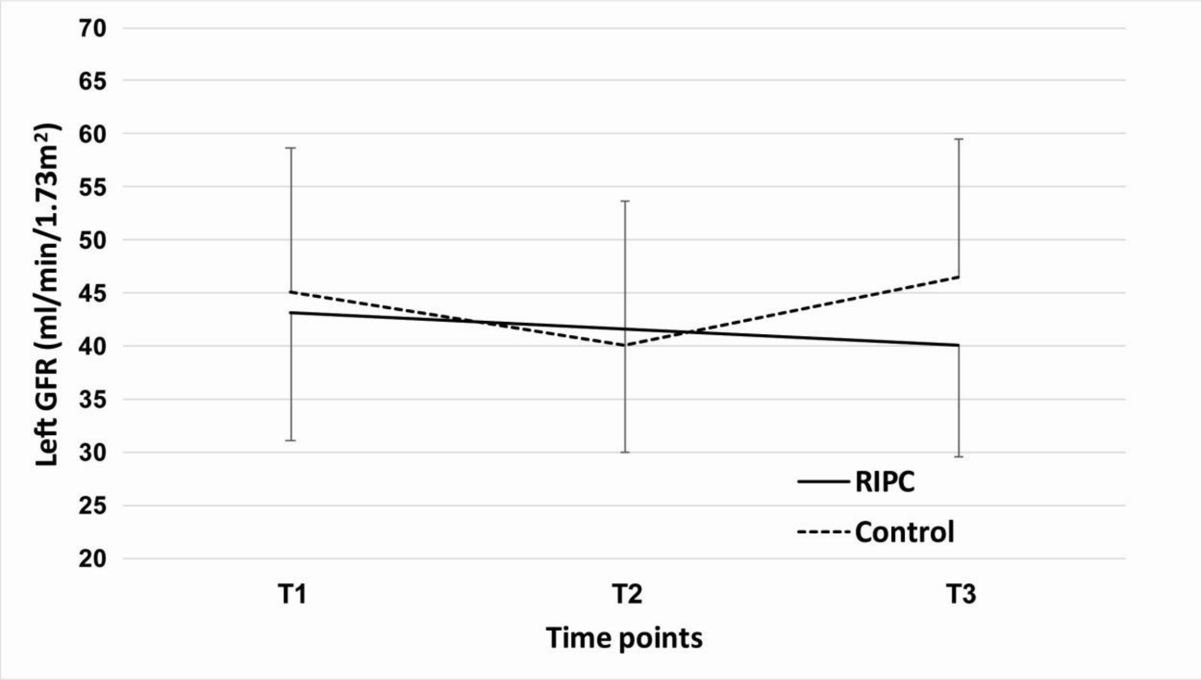

Supplementary Figure S2. Comparison of time dependent change of left glomerular filtration rate measured by 99mTc-DTPA renal scintigraphy between RIPC and control group. The time points were defined as preoperative (T1), 6 month after surgery (T2) and 12~18 month after surgery (T3).

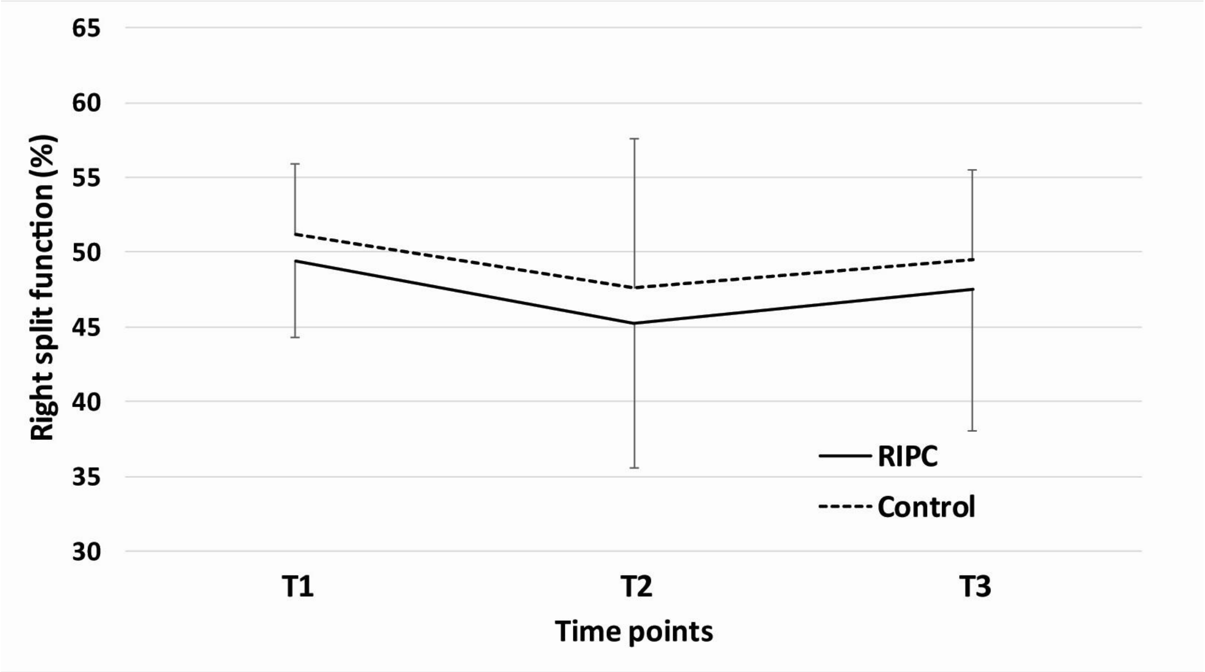

Supplementary Figure S3. Comparison of time dependent change of right split renal function measured by ^{99m}Tc-DTPA renal scintigraphy between RIPC and control group. The time points were defined as preoperative (T1), 6 month after surgery (T2) and 12~18 month after surgery (T3).

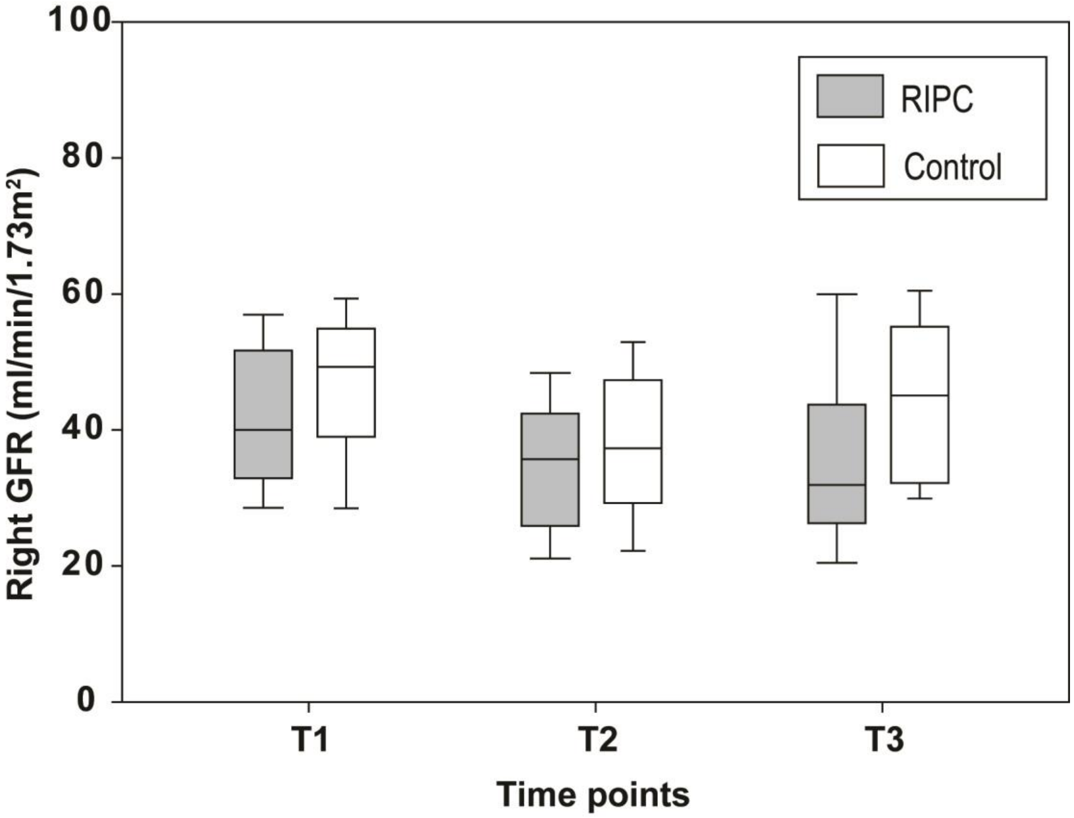

Supplementary Figure S4. Comparison of time dependent change of right glomerular filtration rate measured by ^{99m}Tc-DTPA renal scintigraphy between RIPC and control group. The time points were defined as preoperative (T1), 6 month after surgery (T2) and 12~18 month after surgery (T3).

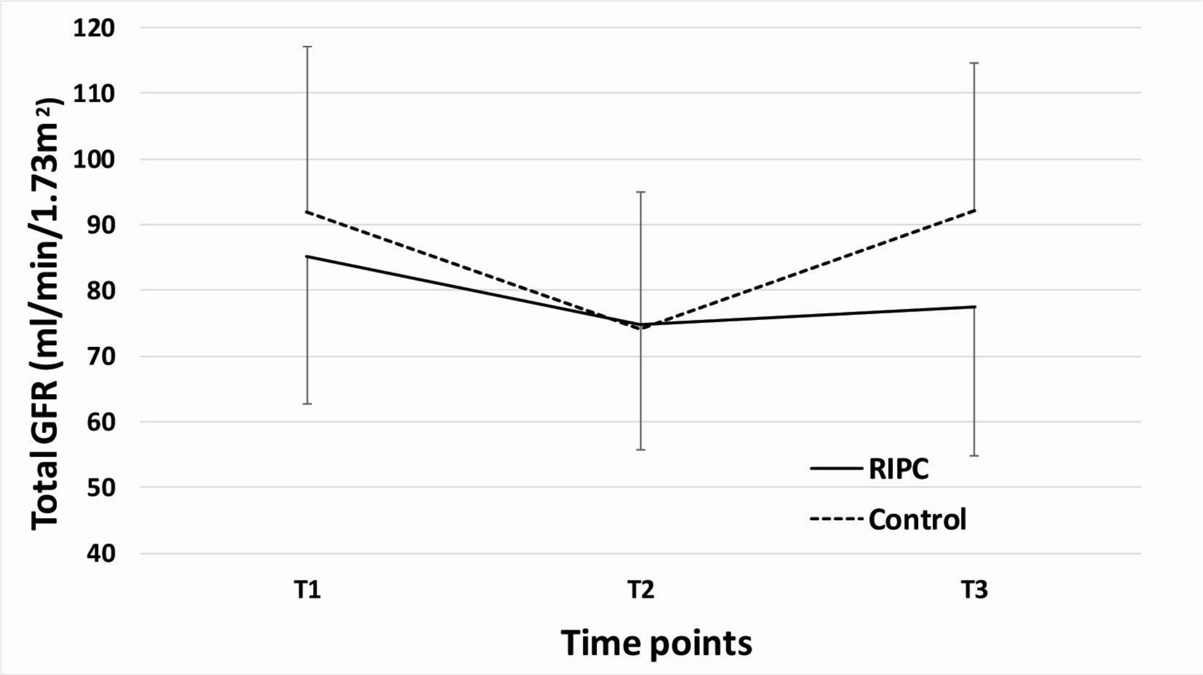

Supplementary Figure S5. Comparison of time dependent change of total glomerular filtration rate measured by ^{99m}Tc-DTPA renal scintigraphy between RIPC and control group. The time points were defined as preoperative (T1), 6 month after surgery (T2) and 12~18 month after surgery (T3).

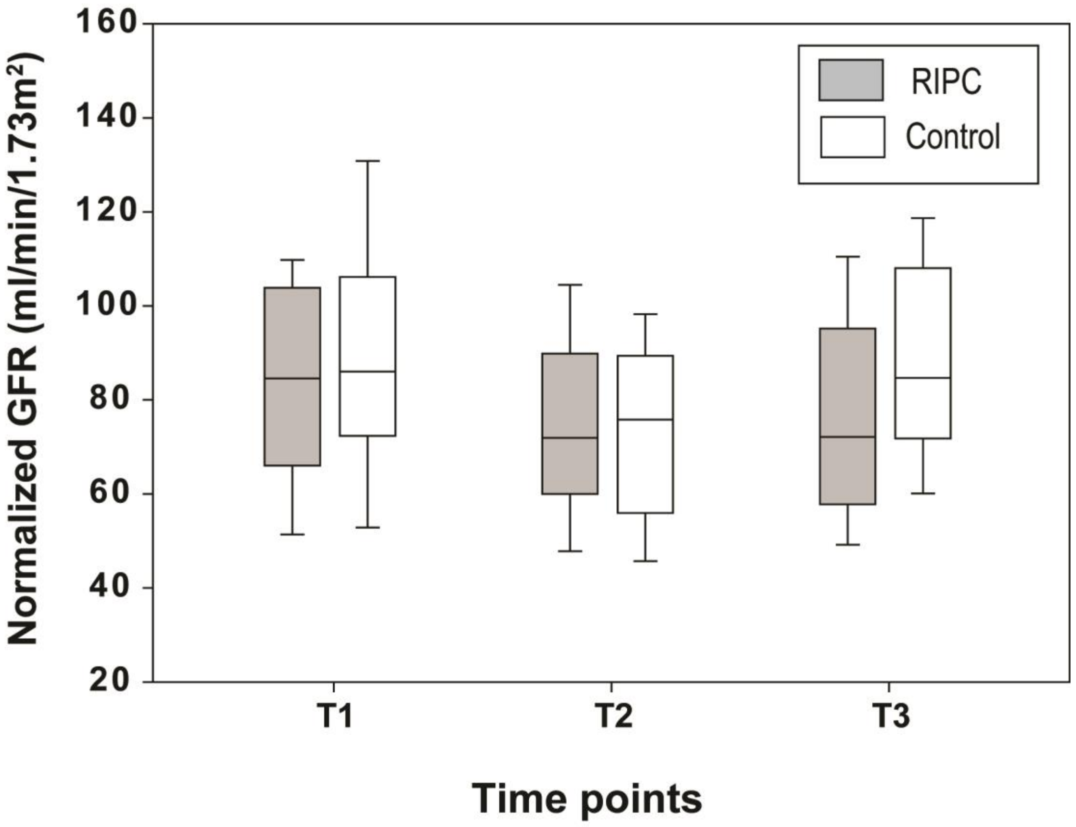

Supplementary Figure S6. Comparison of time dependent change of normalized glomerular filtration rate measured by ^{99m}Tc-DTPA renal scintigraphy between RIPC and control group. The time points were defined as preoperative (T1), 6 month after surgery (T2) and 12~18 month after surgery (T3).

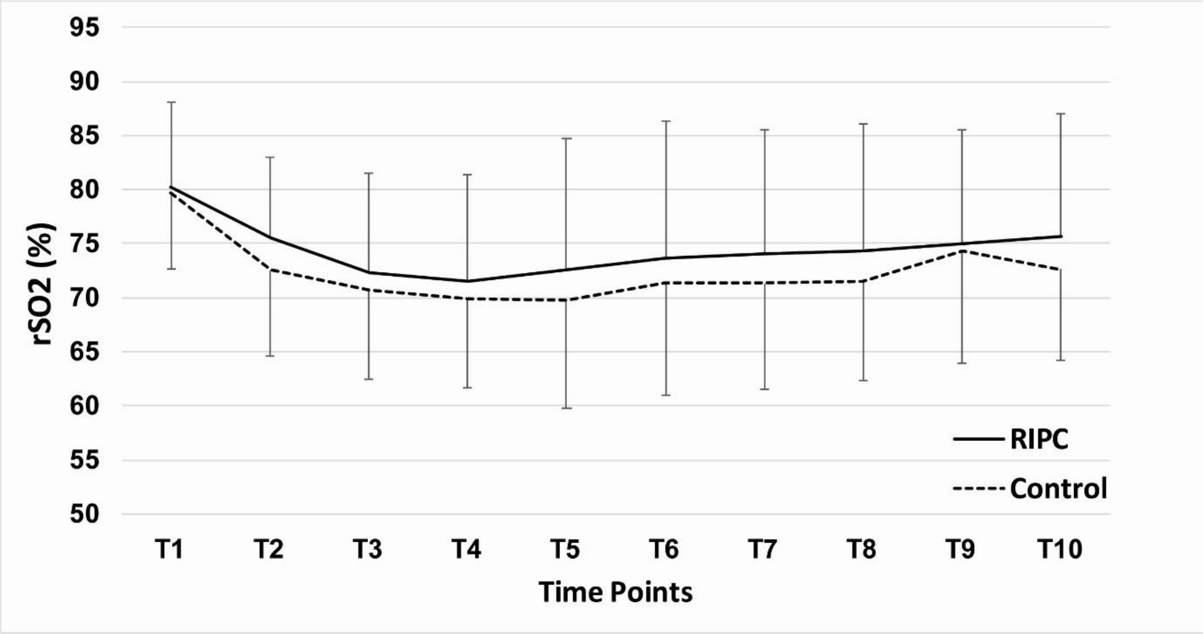

Supplementary Figure S7. Comparison of time dependent change of regional O_2 saturation (rSO_2) during partial nephrectomy between RIPC and control group. The rSO_2 was measured intraoperatively from baseline (T1) to 90 minutes after surgery (T10) for every 10 minutes.

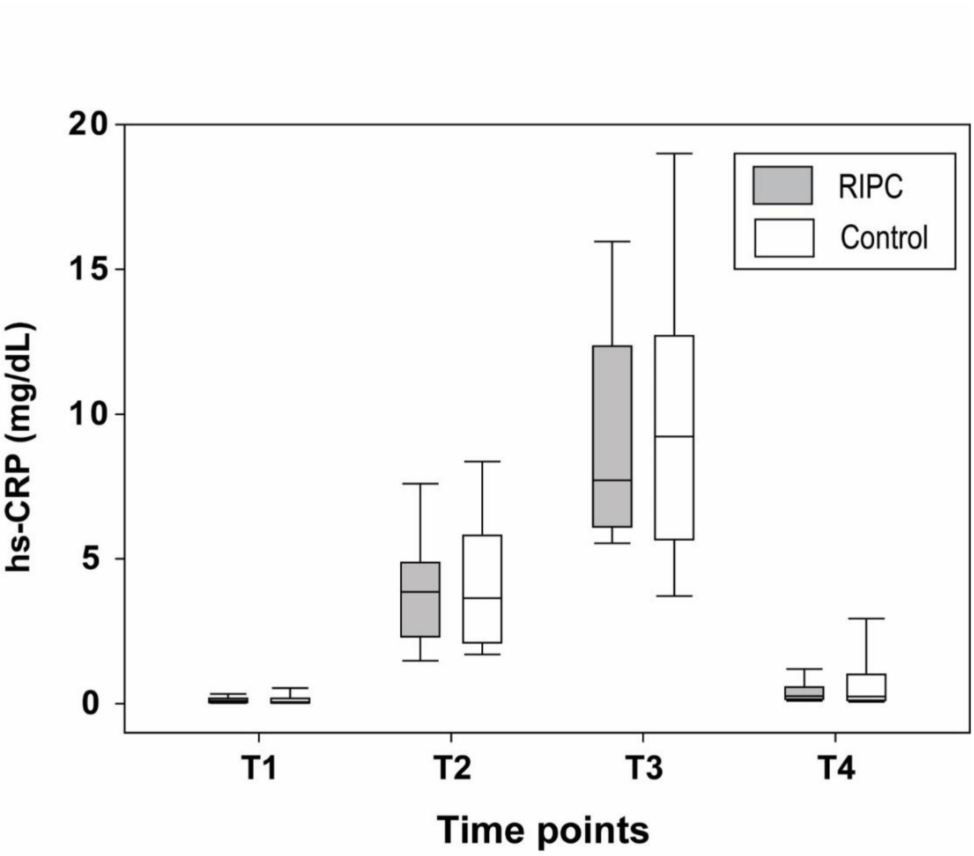

Supplementary Figure S8. Comparison of time dependent change of high sensitive C-reactive protein between RIPC and control group. Time points were defined as preoperative (T1), postoperative day 1 (T2), postoperative day 3 (T3), and two weeks after surgery (T4).

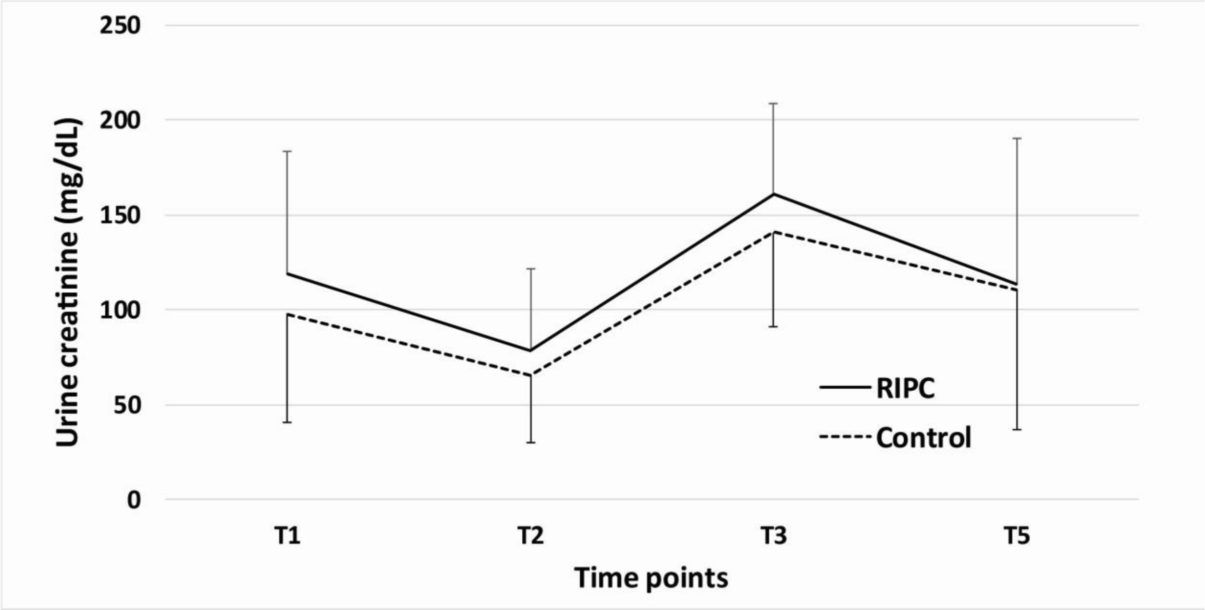

Supplementary Figure S9. Comparison of time dependent change of urine creatinine between RIPC and control group. Time points were defined as preoperative (T1), at post-anesthesia care unit (T2), postoperative day 1 (T3), and two weeks after surgery (T4).

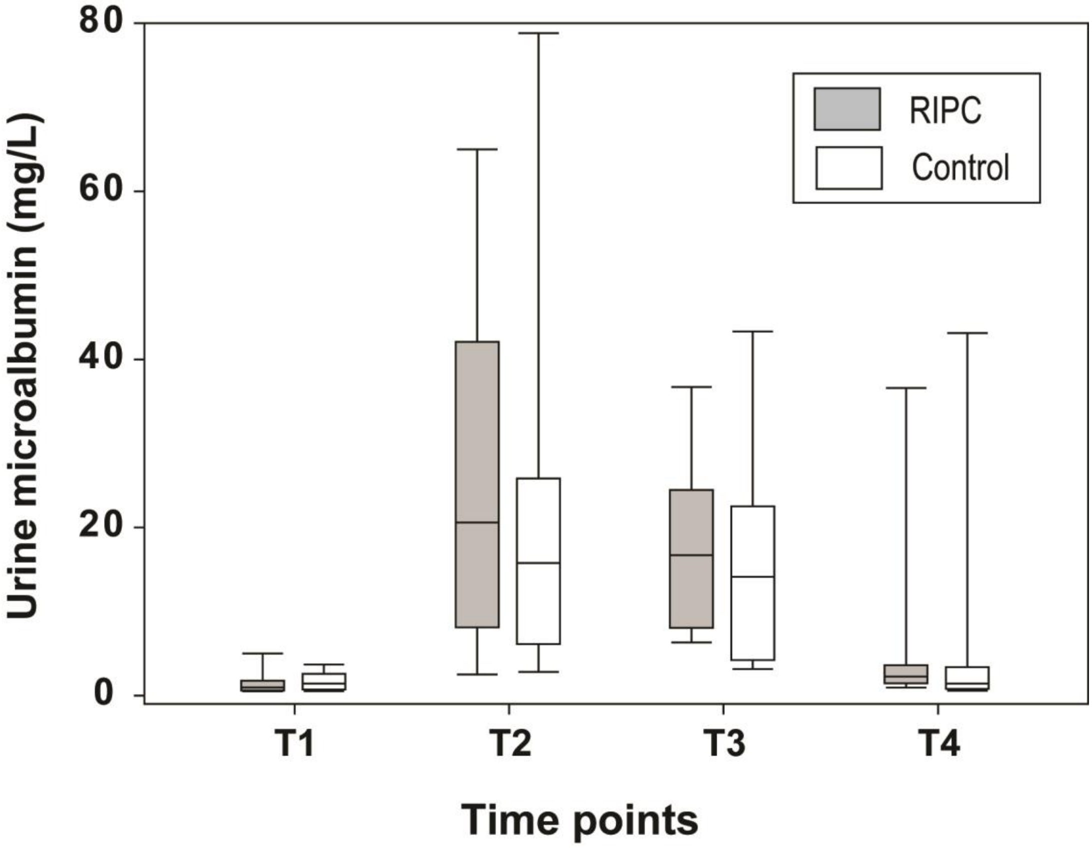

Supplementary Figure S10. Comparison of time dependent change of urine microalbumin between RIPC and control group. Time points were defined as preoperative (T1), at post-anesthesia care unit (T2), postoperative day 1 (T3), and two weeks after surgery (T4).

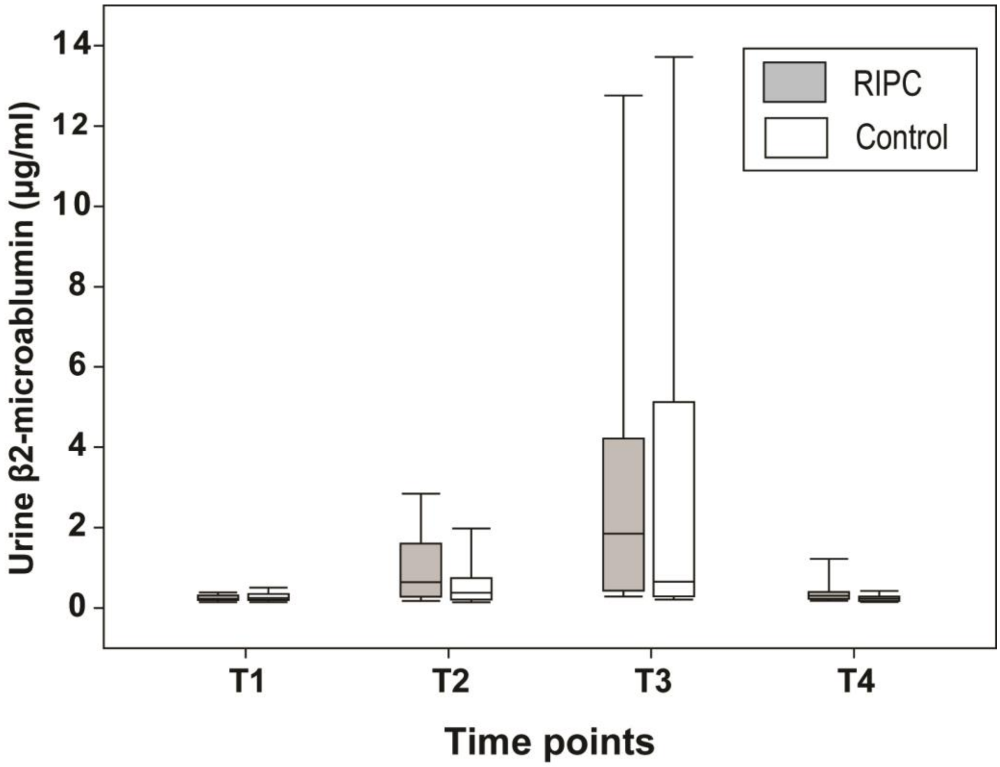

Supplementary Figure S11. Comparison of time dependent change of urine β-2 microglobulin between RIPC and control group. Time points were defined as preoperative (T1), at post-anesthesia care unit (T2), postoperative day 1 (T3), and two weeks after surgery (T4).

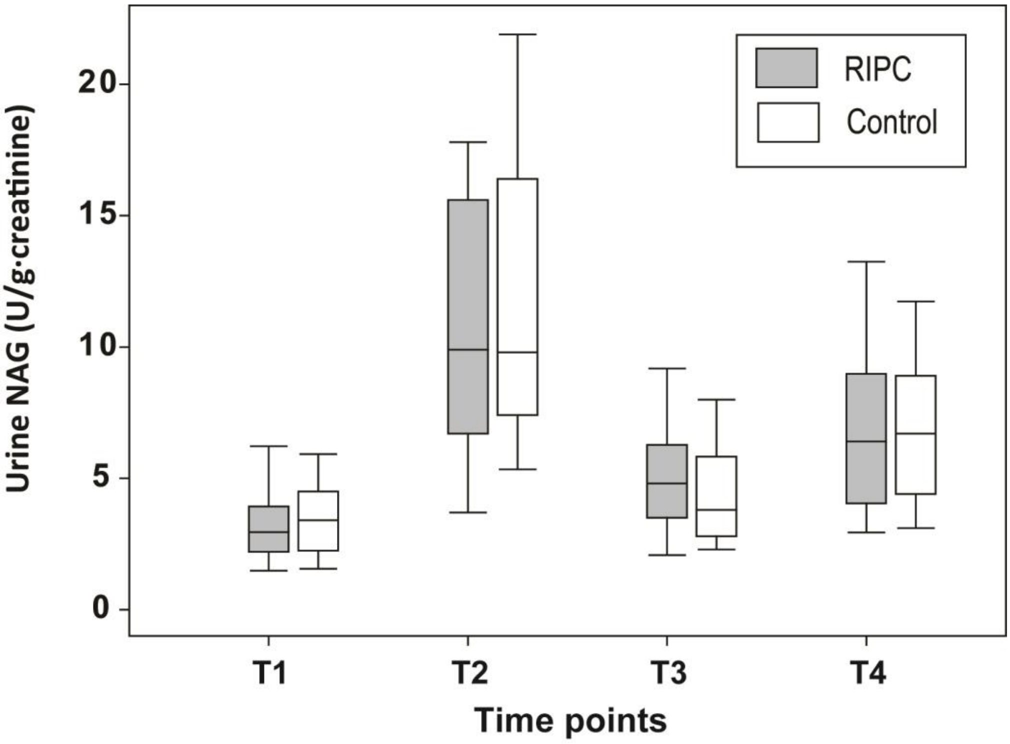

Supplementary Figure S12. Comparison of time dependent change of urine N-acetyl-beta-D-glucosaminidase (NAG) between RIPC and control group. Time points were defined as preoperative (T1), at post-anesthesia care unit (T2), post-operative day 1 (T3), and two weeks after surgery (T4).
